# Skin-to-skin contact in neonatal intensive care units: a narrative review of implementation challenges and strategies within infant- and family-centered care

**DOI:** 10.3389/fped.2026.1869554

**Published:** 2026-06-19

**Authors:** Alicia Montaner-Ramon, Fatima Camba-Longueira, Josep Perapoch-Lopez

**Affiliations:** 1 Neonatology Department, Vall D'Hebron Hospital Universitari, Vall D'Hebron Barcelona Hospital Campus, Barcelona, Spain; 2 NIDCAP Barcelona Training Center – Vall D'Hebron and Dr. Josep Trueta Hospitals, Barcelona, Girona, Spain; 3 Pediatrics Department, Hospital Universitari de Girona Doctor Josep Trueta, Girona, Spain

**Keywords:** skin to skin contact, kangaroo mother care, infant-and family-centered developmental care, neurodevelopmental care, prematurity, neonatal intensive care

## Abstract

Skin-to-skin contact (SSC) is a key component of infant- and family-centered developmental care in neonatal intensive care units (NICU) and is associated with improved clinical and neurodevelopmental outcomes. However, SSC implementation in NICUs remains highly variable, often limited by perceived barriers and uncertainties regarding safety. This review aims to identify these barriers and summarize strategies to support the safe and consistent integration of SSC into routine neonatal care. Current evidence identifies barriers at multiple levels, including clinical factors (extreme prematurity, respiratory support, hemodynamic instability, maternal conditions), organizational constraints (lack of standardized protocols, limited staffing, variability in professional training), and emotional challenges (parental stress, insufficient guidance, and inconsistent support from healthcare professionals). Although concerns about safety—such as accidental device dislodgement or clinical instability—are frequently cited, current evidence does not support withholding SSC when appropriate monitoring, preparation, and positioning are ensured. Recent studies highlight that interventions like multidisciplinary professional training, simulation-based learning, institutional protocols, standardized safety checklists, algorithms for eligibility and structured parental education, are effective to reduce variability and support safe implementation of SSC. These interventions have been associated with increased SSC rates, improved parental engagement, and enhanced alignment with developmental care practices. Addressing these multilevel barriers is essential to promote the safe, equitable, and sustainable implementation of SSC across diverse neonatal care settings.

## 1 Background

Preterm birth remains a critical global health challenge, with 13.4 million babies born prematurely in 2020—approximately one in ten births worldwide (1). Given that complications arising from prematurity continue to be the leading cause of death in children under five, identifying high-impact interventions to reduce them is crucial (1, 2). In this context, Kangaroo Mother Care (KMC), characterized by early, continuous, and prolonged skin-to-skin contact (SSC), has evolved from an alternative for resource-limited settings to a gold-standard, evidence-based pillar of neonatal medicine. This shift is further reinforced by the latest World Health Organization (WHO) guidelines, which now recommend initiating KMC immediately after birth, redefining it as a fundamental component of essential neonatal care (1, 3–6).

The clinical superiority of SSC over conventional incubator care is unequivocal. Meta-analyses demonstrate a 33% to 40% reduction in neonatal mortality, along with significantly lower rates of sepsis, hypothermia, and hypoglycemia (1, 4, 7). Beyond immediate survival, SSC acts as a neuroprotective intervention that optimizes physiological stability, improves sleep organization, and promotes long-term neurodevelopment (1, 4, 8, 9). Moreover, it stands out as a highly cost-effective strategy that does not require the high-tech infrastructure or expensive equipment typical of other neonatal intensive care treatments. In addition, early initiation of SSC has been shown to increase its overall duration throughout the hospitalization period; therefore, starting this intervention as soon as possible should be considered a clinical priority in neonatal units (10, 11). The benefits for parents equally justify the importance of its implementation: SSC attenuates postpartum depression and stress, fosters early bonding, and is a key factor in the success of exclusive breastfeeding (7, 12).

Despite this compelling evidence, the universal implementation of SSC in Neonatal Intensive Care Units (NICUs) remains inconsistent across both high-income and low-to-middle-income countries; in certain high-technology environments, adoption rates for early KMC are reported as low as 21.2% (9, 13, 14). This inconsistency is not a failure of evidence, but a reflection of a complex interplay between structural, clinical, and sociocultural barriers (12).

Structural and environmental constraints are often prominent, particularly in open-bay NICUs that lack privacy, adequate space, ergonomic furniture, and bedside equipment to support prolonged SSC (1, 12). In parallel, healthcare professionals frequently express concerns regarding the safety of unstable infants, including the risk of accidental extubation or catheter dislodgement, which may delay or limit SSC initiation (12, 15, 16). However, accumulating evidence indicates that SSC is safe and feasible even in extremely preterm infants and in those with umbilical catheters or requiring mechanical ventilation (9, 15, 17, 18).

At the family level, parental participation may be further hindered by physical, logistical, and socio-cultural factors. Postpartum discomfort, particularly following cesarean delivery, together with lack of accommodation near hospital, and the resulting transport challenges, can restrict parental presence. Additionally, cultural practices such as the postpartum confinement period in some contexts may influence family expectations and delay early engagement in SSC (9, 17, 18).

Under the Infant- and Family-Centered Developmental Care (IFCDC) paradigm, the focus is shifting towards “Zero Separation.” This model transcends the view of parents as mere visitors, integrating them as primary and irreplaceable caregivers. To overcome the current implementation gap, NICUs must move from considering SSC as an optional adjunct to a core and non-negotiable clinical practice (12, 19–22).

This implementation-focused narrative review explores barriers and practical strategies related to SSC implementation in NICUs. Unlike previous reviews, which primarily focused on the physiological benefits of kangaroo mother care or SSC, or on implementation in resource-limited settings, this review specifically discusses challenges and implementation approaches across different neonatal care contexts, including high-complexity NICUs. In addition, it integrates clinical safety, organizational culture, neonatal architecture, parental psychosocial factors, equity, and family-centered care perspectives into a thematic discussion of SSC implementation.

## 2 Methods

This manuscript was developed as an implementation-focused narrative review aimed at exploring barriers to the initiation and implementation of SSC in NICUs, as well as identifying practical strategies to facilitate its integration into routine neonatal care within an IFCDC framework.

### 2.1 Literature search strategy

A non-systematic literature search was conducted using PubMed/MEDLINE and Google Scholar databases between January and March 2026. The search included combinations of the following terms: “skin-to-skin contact”, “kangaroo mother care”, “kangaroo care”, “neonatal intensive care unit”, “implementation”, “barriers”, “facilitators”, “family-centered care”, “single-family room”, “structural barriers”, “socio-economic barriers”, “prematurity”, “extremely preterm infants”, “mechanical ventilation”, “high frequency oscillatory ventilation”, “umbilical catheters”, “simulation-based training”, “parental involvement”, “maternal mental health”, and “trauma-informed care”.

In addition, OpenEvidence was used as a supplementary clinical search tool to support targeted exploration of specific implementation- and safety-related questions and to help identify additional relevant publications.

The search prioritized publications from the last 10 years. However, seminal publications and older studies considered essential for contextual understanding or historical relevance were also included.

Reference lists of selected articles were additionally screened to identify further relevant publications.

### 2.2 Eligibility criteria and study selection

Original studies, systematic reviews, meta-analyses, qualitative studies, implementation studies, consensus documents, clinical guidelines, and quality-improvement publications related to SSC in neonatal care were considered eligible for inclusion.

Conference abstracts without full-text availability, isolated case reports, and studies not related to neonatal SSC implementation were excluded.

The literature was reviewed independently by two authors, who discussed the relevance and contribution of each publication according to the objectives of the review. Discrepancies were resolved by consensus.

### 2.3 Data synthesis

The selected literature was synthesized narratively and organized into thematic domains addressing structural and organizational barriers, perceptions of clinical safety and stability, barriers related to healthcare professionals and the institution, and parental and sociocultural barriers, as well as implementation strategies for each domain that support the integration of the SSC. Given the narrative and implementation-focused nature of this review, a formal quality appraisal or risk-of-bias assessment and meta-analysis were not performed, and article selection was based on thematic relevance and the authors' appraisal of the literature.

## 3 Understanding the barriers to the implementation of skin-to-skin contact in neonatal intensive care units

**Figure 1:**
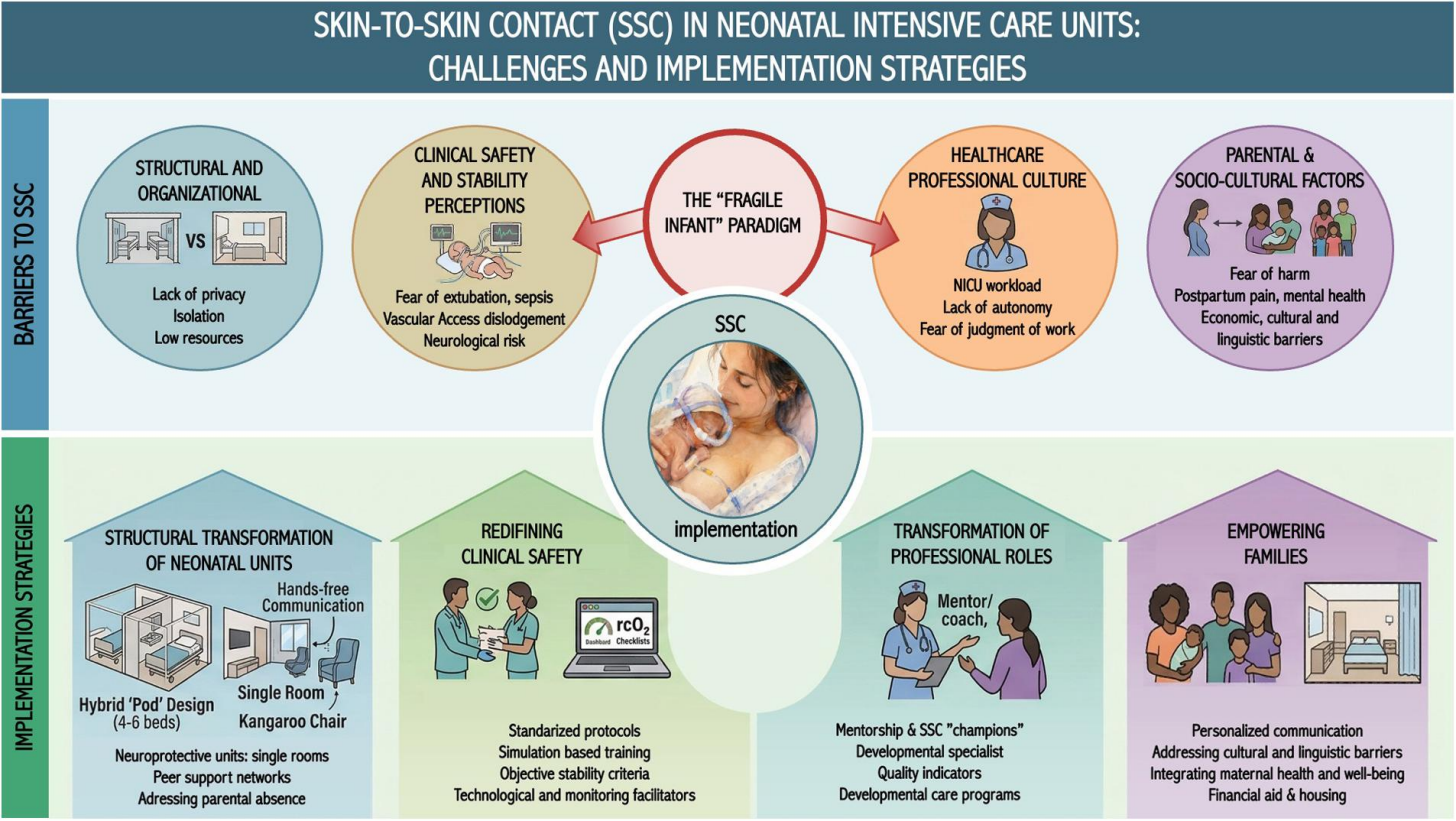
Graphical summary of challenge and strategies for skin-to-skin contact implementation in neonatal care units.

The integration of SSC as a routine practice in NICUs remains a complex challenge, shaped by interacting structural, human, and system-level factors. Despite the strength of the clinical evidence, its implementation remains far from universal. This gap between evidence and practice suggests that SSC is not merely a clinical intervention, but a paradigm shift that requires alignment between parental readiness, professional autonomy, and institutional support. Depending on how these elements are configured, they may either enable or hinder its adoption. The main barrier domains, together with potential implementation strategies, are summarized in Table 1 and illustrated in Figure 1. The implementation strategies presented include a combination of evidence-supported interventions, emerging approaches, and expert-opinion proposals identified throughout the reviewed literature.

**Table 1:** Conceptual thematic synthesis of the main barriers, implementation strategies, and current knowledge gaps identified throughout the narrative review.

Barrier domain	Main barriers	Potential implementation strategies	Evidence/feasibility	Knowledge gaps
Structural and organizational barriers	Lack of privacy in open-bay NICUs Limited ergonomic space Inadequate furniture Sensory overstimulation Concerns regarding SFR isolation and safety	Ergonomic SSC chairs Privacy screens Hands-free communication systems Parent guidance tools Peer-support activities Hybrid room models Gradual transition toward SFR design when feasible	Moderate evidence; several low-cost strategies feasible across settings	Optimal balance between privacy, stimulation, safety, and equity across NICU models
Clinical safety and stability perceptions	Fear of accidental extubation, airway obstruction, catheter displacement, infections, intraventricular hemorrhage, infant falls. Exclusion of extremely preterm infants or infants with HFOV.	Standardized eligibility and transfer protocols Objective stability criteria Simulation-based multidisciplinary training Use of positioning/support devices Monitoring technologies (e.g., NIRS) Digital parent education and SSC tracking tools/apps	Moderate-to-high evidence for safety in many high-risk populations	SSC in infants receiving HFOV, pleural drains, or at the limit of viability (22–24 weeks)
Barriers related to healthcare professionals and the institution	SSC perceived as secondary task Understaffing and high workload Need for two trained professionals for transfers Limited nursing autonomy Lack of protocols or institutional prioritization	SSC champions Staff education: Simulation-based training Standardized SSC protocols Interprofessional collaboration Institutional prioritization (SSC-related quality indicators) Staffing and workflow optimization	Moderate evidence; highly feasible and scalable	Optimal staffing models, sustainability of training programs, implementation in low-resource settings
Parental and sociocultural barriers	Fear of harming the infant Parental anxiety and trauma-related responses Maternal pain or illness Language and cultural barriers Socioeconomic difficulties Limited parental presence or reduced involvement	Trauma-informed care Psychological support Culturally adapted communication; interpreters Peer-support and community-building programs Inclusion of fathers/non-birthing parents and other relatives Family education tools Financial/logistical support programs	Moderate evidence; feasibility depends on local resources	Effective trauma-informed interventions, strategies to support families with limited NICU presence, long-term impact of peer-support models

SFR, Single-family rooms; SSC, Skin-to-skin contact; HFOV, high-frequency oscillatory ventilation; NICU: Neonatal intensive care unit.

### 3.1 Structural and organizational barriers

The physical architecture of NICUs is a determining factor in the care provided and the degree of parental involvement in their children's care (23). Traditional “open room” designs, while optimized for direct staff supervision, often limit the initiation of SSC due to a lack of privacy, high exposure to stressful sensory stimuli, and insufficient ergonomic space around the incubator (1, 9, 12, 24, 25). In this context, a significant architectural debate has emerged contrasting the traditional open-bay design with the single-family room (SFR) model (23, 26, 27).

Single-family rooms (SFRs) have been associated with increased SSC duration, reduced parental stress, and improved outcomes such as breastfeeding rates and weight gain (23, 25, 28, 29). However, there is significant concern surrounding excessive sensory deprivation which may diminish the essential auditory stimuli necessary for regional cortical folding and functional language maturation (23, 30). Indeed, some longitudinal assessments link this sensory deprivation to lower language scores at two years of age (30, 31).

Nevertheless, other studies have reported better cognitive and linguistic outcomes in NICUs with SFR (31, 32). This discrepancy suggests that neurodevelopmental outcomes may depend less on room design itself than on the quality of parental presence, interaction, and sensory stimulation provided to the infant. Therefore, while SFRs may facilitate SSC and family-centered care, they should not be considered a prerequisite for SSC implementation (27, 29, 30, 32).

This architectural shift also introduces important safety concerns, as both staff and families often perceive a greater risk in SFRs due to the loss of direct visibility (26, 27). Healthcare professionals often worry that reduced vigilance may delay the detection of acute clinical events such as accidental extubations or device dislodgement (26, 27), while parents may feel anxious when their baby is left alone in the room. In addition, they often exhibit a fear of handling their fragile baby without the immediate proximity of nursing staff, especially in the early stages of admission, when they lack the necessary experience and confidence (18, 23, 33).

Furthermore, the transition to private spaces presents social challenges (25, 27). While the privacy of a SFR is highly valued by families, both parents and staff often report a sense of isolation, missing the informal peer support that naturally occurs in shared spaces (23, 25–27).

In addition, this architectural change has significant implications for team dynamics and functioning. The transition to physically separate rooms reduces opportunities for observational or “vicarious” learning, which is particularly relevant for less experienced staff who traditionally acquire skills by observing more experienced professionals and interacting with colleagues. At the same time, working in isolated rooms can increase perceptions of professional isolation and require more structured communication strategies (26, 27, 34).

Uncertainties persist regarding the optimal balance between privacy, sensory stimulation, safety, and feasibility in different organizational models.

### 3.2 Clinical safety and stability perceptions

Perhaps the greatest limitation to SSC implementation in neonatal care is the widespread perception of the “fragile baby” (19, 35), in which some clinical situations and technological support are often viewed as contraindications to SSC, and incubator rest is often prioritized over SSC (19, 35, 36). Where the safety of the newborn is obviously paramount, individual beliefs about safety frequently take precedence over evidence-based guidelines (12, 19, 36).

Surveys of healthcare providers consistently highlight significant anxiety regarding the safety of SSC in critically ill infants (36, 37). In this context, professional concerns regarding SSC generally focus on five main areas. However, current evidence suggests that SSC is safe and feasible even in highly complex neonatal populations when standardized protocols and trained staff are available.

- **Respiratory safety and extreme prematurity:** Concerns regarding accidental extubation or respiratory instability remain among the main barriers to SSC implementation. Nevertheless, studies have shown that SSC does not increase accidental extubation or airway complications when performed by trained personnel and may provide physiological stabilization comparable or superior to incubator care even in extremely preterm infants and during planned extubation (12, 16, 35, 38–40).
- **Vascular access:** Concerns persist regarding displacement or bleeding associated with umbilical catheters or peripherally inserted central catheters (PICCs) (15, 35, 41). However, the available evidence suggests that performing SSC does not appear to increase the risk of displacement, bleeding, or accidental removal during SSC when appropriate positioning and monitoring protocols are followed (35, 39, 42–44).
- **Infection control:** In some clinical settings, parental presence may still be perceived from a “biosecurity” perspective, with concerns that personal items may be vectors for nosocomial sepsis (1, 9, 14, 45). Nonetheless, SSC has been associated with lower rates of sepsis and infection-related mortality, likely through promotion of protective maternal microbiota colonization and reducing stress-induced immunosuppression, provided that rigorous hand hygiene measures are maintained (5, 43, 45–48).
- **Neurological risk:** Specific controversy persists regarding intraventricular hemorrhage (IVH). Although there is a historical concern that head rotation during SSC may impede jugular venous drainage and increase the risk of IVH, this is not supported by recent large-scale data, and early initiation (Day 0–1) has not been associated with a higher incidence of severe IVH (Grades III-IV). In fact, the physiological stability and reduced blood pressure surges provided by SSC may act as a neuroprotective mechanism (35, 43, 45, 49).
- **Infant falls and positioning accidents:** A significant fear, though often underreported, is the risk of the infant falling during SSC, particularly when parents are asleep or if the baby is extremely small. This concern can lead to restrictive policies or the need for constant, individualized professional supervision, which may be difficult to sustain in understaffed units (33, 50).

Important evidence gaps persist regarding SSC in infants at the limit of viability (22–24 weeks of gestation) and in those receiving high frequency oscillatory ventilation (HFOV) or devices such as pleural drains, as these populations continue to be underrepresented in clinical studies. However, preliminary evidence and recent quality improvement initiatives suggest that SSC can be feasible and safe even in these complex clinical situations when appropriate protocols, simulation-based training, and specialized support measures are available (6, 38, 39, 41, 44).

Taken together, these findings highlight the need for standardized transfer protocols, simulation-based training, and multidisciplinary approaches that reduce variability in practice, which still depends excessively on individual professional perceptions rather than standardized criteria.

### 3.3 Barriers related to healthcare professionals and the institution

The transition of SSC from an optional intervention to an essential treatment is strongly influenced by the professional culture in NICUs, institutional priorities, and staffing conditions (12, 36, 51).

SSC is often perceived as an additional activity rather than a fundamental treatment, competing with technical and pharmacological tasks considered to be of higher priority (12, 49, 52, 53). This perception is further reinforced in understaffed units, where SSC is often relegated to a secondary role behind other daily tasks, particularly in high-acuity settings requiring complex coordination (1, 12, 24, 49). As a result, staffing conditions and workload become major determinants of SSC feasibility, especially in resource-limited settings. International variations in nurse-to-patient ratios may further contribute to inequities in SSC implementation across different healthcare systems (1, 24).

Professional hierarchies and organizational culture may further limit SSC integration into routine care. In some settings, nursing staff feel unable to initiate SSC without explicit medical authorization, transforming SSC into a physician-dependent intervention (13, 52). At the same time, the shift toward family-centered care could generate anxiety among professionals, who may fear adverse events or feel uncomfortable providing care under parental observation. Furthermore, deficiencies in staff training, lack of standardized protocols, and limited prioritization by institutions—such as the absence of SSC-related quality indicators in NICUs—contribute to persistent variability in practice across professionals and units (14, 25, 27).

Addressing these barriers requires not only education and simulation-based training, but also a cultural and organizational shift that recognizes SSC as a standard and non-negotiable component of neonatal care.

### 3.4 Parental and sociocultural barriers

For many families, the transition from passive “visitors” to active “primary caregivers” is limited by a complex combination of physical, psychological, social, and cultural barriers. The highly medicalized environment together with the early separation that frequently accompanies NICU admission may hinder bonding and delay parental involvement in SSC (12, 18, 24, 31, 33, 54, 55).

- **Internal barriers:** The most widespread internal barrier is the fear of harming an infant perceived as extremely fragile, particularly during the first attempts at contact. Parental anxiety may be reinforced by inconsistent messages from healthcare professionals, maternal guilt, and hypervigilance toward monitors and medical devices, leading parents to focus more on technology than on interaction with their infant (1, 9, 12, 17, 18, 33, 41). In this context, parental psychological distress, depression, and trauma-related responses may directly reduce SSC participation through avoidance behaviors and difficulties engaging with the NICU environment.
- **Maternal health:** Maternal health is a critical determinant of SSC frequency and continuity. Postpartum pain, particularly after cesarean sections, and maternal mental health problems such as depression affect nearly half of the mothers in the NICU, reducing their physical and emotional capacity to remain continuously present in the NICU (1, 5, 9, 18, 24, 33, 56).
- **Economic factors:** Economic factors, such as long travels to the hospital, lack of paid parental leave in some settings, meal costs, and the challenges of caring for siblings at home, create a significant financial and logistical burden for many families, which may limit parental presence and reduce SSC continuity over time (1, 9, 17, 18, 24, 31, 33).
- **Cultural barriers:** Cultural and linguistic barriers may contribute to inequities in SSC implementation. Limited proficiency in the primary language of the healthcare system, cultural traditions surrounding postpartum confinement, social stigma related to prematurity, and fear associated with uncertain neonatal outcomes may all reduce parental involvement and delay the initiation or decrease the daily duration of SSC (1, 5, 13, 57–59).
- **Diversity in family structures and psychosocial vulnerability:** In some settings, a significant gap still exists regarding parental involvement and the inclusion of what are considered non-normative family units. Prevailing gender norms may unintentionally exclude fathers and non-birthing caregivers from active participation in SSC and neonatal care, particularly during periods in which mothers are temporarily unavailable due to postnatal recovery, cesarean delivery, or obstetric complications (9, 24, 52, 60). Furthermore, many NICUs may struggle to address the specific needs of families dealing with severe mental health conditions, psychosocial vulnerability, or substance use disorders (12, 61, 62).

Taken together, these barriers contribute to significant variability and inequity in SSC implementation across NICUs. As a result, access to SSC often depends not only on the infant's clinical condition but also on contextual factors such as institutional culture, professional attitudes, and the availability of psychosocial and family support resources. Addressing this disparity requires moving beyond individual changes and adopting a more standardized and systemic approach that ensures the systematic integration of SSC as a fundamental component of neonatal care.

## 4 From barriers to action: implementation strategies for skin-to-skin contact in neonatal intensive care units

Recognizing that barriers to SSC exist at various levels within neonatal units, implementation strategies should be equally comprehensive and coordinated. Integrating SSC into routine neonatal practice requires approaches that simultaneously address structural, safety, and training aspects for both professionals and families. While isolated interventions can increase SSC implementation at specific times and are better than no intervention, efforts should focus on creating sustainable strategies that support SSC as a routine component of care over time (12, 19, 46, 52, 63).

Reducing variability in SSC implementation between institutions, healthcare systems, and individual professionals should be considered a central objective of neonatal care. However, important differences in starting point, resources, staffing, infrastructure, and organizational culture make universal implementation particularly challenging across diverse NICU settings. Establishing consistent guidelines, supported by structured training and institutional commitment, is essential to promoting access to SSC regardless of the setting. Equally important is fostering a shared understanding of SSC among healthcare professionals and families, promoting parental participation as a central component of neonatal care rather than as a complementary intervention. Through coordinated multi-level strategies, SSC may progressively transition from a variable practice dependent on local culture and individual beliefs into a standard component of neonatal intensive care.

Although multiple implementation strategies have been proposed, uncertainties remain regarding which combinations of interventions are the most effective, sustainable over time, and adaptable across different healthcare settings and resource levels.

### 4.1 Structural transformation of neonatal units: designing environments that support family presence and neuroprotective care

The physical and organizational design of NICUs should not be viewed merely as a barrier to SSC implementation, but rather as an opportunity to facilitate parental presence, neuroprotective care, and family participation.

#### 4.1.1 Unit design

The transition towards single rooms may represent one possible strategy for increasing parental presence and involvement particularly in high-resource settings. SFRs have been associated with increased parental presence, longer SSC duration, and greater participation in neonatal care. However, SFR infrastructure is not universally available or inherently superior in all contexts, and current evidence suggests that the quality and continuity of parental involvement may be more important than room design itself (17, 23, 25, 64, 65).

Although private rooms may facilitate privacy, comfort, and parental autonomy, they may also introduce challenges related to staff visibility, communication, and social isolation (23, 27). To address these challenges, several strategies have been proposed. Hands-free communication devices and family guidance tools (such as videos, interactive explanations of the benefits of SSC, and instructions on how to contact staff) can help reduce parental anxiety and boost their confidence to participate in the newborn's care (17, 27, 34).

NICU environments should aim to facilitate safe SSC, continuous family presence, and neurodevelopmental care, regardless of the architectural model. Several low-cost, adaptable interventions can improve SSC implementation even in open-bay units, such as ergonomic chairs, privacy screens, family-friendly spaces, and structured family counseling tools (17, 23, 24).

#### 4.1.2 Peer support and community building

To minimize the social isolation inherent in NICU admissions, units should foster community among families (25, 27, 66). Organizing recreational activities and support groups led by experienced parents may represent useful strategies for coping with the emotional trauma of NICU admission and building bonds among families (66, 67). Furthermore, they can facilitate the exchange of practical advice and success stories, strengthening the resilience needed to endure prolonged hospital stays and promoting the presence of families with their children, thus facilitating SSC (62, 66, 67).

#### 4.1.3 Addressing parental absence

Little is known about how to optimize SSC implementation in infants whose families cannot be present for longer periods due to socioeconomic, geographic or family-related barriers (12, 18, 31, 68, 69). Long travel distances, work obligations, lack of parental leave, or the need to care for other children may significantly limit parental presence, particularly during prolonged admissions.

In this context, ambitious proposals such as the temporary re-enrollment of siblings in schools near the hospital or the creation of units where the entire family can cohabit have been proposed as potential strategies for maintaining family unity and facilitating parental presence, although these approaches remain largely unexplored and may not be feasible in many healthcare settings (18, 31).

In the absence of such policies, alternative strategies may help reduce the negative impact of parental absence. Preliminary evidence suggests that involving other family members (especially grandmothers) in SSC may increase the total daily duration (42, 70, 71). Volunteer holding programmes have also been proposed as a way to provide human contact and sensory stimulation for infants with limited family presence, although these interventions cannot replace parental bonding and evidence regarding their long-term impact and feasibility remains limited (72).

In this context, some authors have proposed hybrid unit designs, with family rooms, but also shared rooms with few beds, which may help reduce sensory deprivation for these infants with limited parental presence. This alternative, however, could pose an ethical conflict, as it may imply unequal access to more private and family-centered environments. Given the limited and sometimes conflicting evidence, the optimal balance between privacy, stimulation, and equity remains an open question requiring further research (23, 30, 73).

### 4.2 Redefining clinical stability: strategies to promote skin-to-skin contact in fragile infants and support its safe implementation

To avoid individual contraindications based on subjective perceptions of “fragile infant,” neonatology societies and NICUs should adopt standardized, evidence-based approaches that support SSC implementation even in highly complex clinical situations.

#### 4.2.1 Standardization of SSC protocols

The availability of standardized SSC protocols is essential to reduce variability in practice and ensure equitable access to SSC across professionals and institutions (19, 37, 56).

Clearly defined eligibility criteria and transfer procedures may help reduce fears regarding accidental extubation, catheter displacement, and cardiorespiratory instability, ensuring that SSC decisions are based on evidence rather than individual perceptions of safety (12, 19, 35, 36).

Furthermore, defining objective stability criteria ensures that readiness for SSC is determined by the newborn's physiological condition, rather than by the mere presence of devices or technological support, guaranteeing that even the most premature infants are not excluded solely based on their gestational age (6, 16, 38, 39, 41, 43, 74).

However, important evidence gaps remain regarding SSC implementation in infants at the limit of viability, those receiving HFOV, and patients with complex devices such as umbilical arterial catheters or pleural drains (38, 39, 53, 75).

#### 4.2.2 Simulation-based training (SBT) and skills development

The high proportion of professionals feeling discomfort performing SSC in ventilated or critically ill infants indicates a critical need for structured, hands-on training (36, 37). Simulation-based training (SBT) allows multidisciplinary teams to safely practice SSC transfers and management of high-risk situations, including infants receiving HFOV or carrying central catheters. Beyond technical skill acquisition, SBT may improve professional confidence, promote a shared understanding of SSC as a stabilizing intervention, and standardize perceptions of risk across different professional groups (15, 36, 37, 76, 77).

Interprofessional training involving neonatologists, nurses, respiratory therapists, nursing assistants, obstetric teams, and delivery room staff may be particularly important to support SSC implementation throughout the neonatal care pathway (12, 19, 41, 52, 64).

Although SBT appears effective in improving professional competence and confidence, uncertainties remain regarding the optimal frequency, intensity, and sustainability of training programs. Furthermore, training must be tailored to available resources and consider newly hired staff, who in some settings are constantly being replaced.

#### 4.2.3 Facilitator devices: monitoring equipment, furniture, garments and digital tools

Technology should support SSC implementation, rather than hindering its feasibility. Monitoring tools such as near-infrared spectroscopy (NIRS) to monitor regional cerebral oxygenation (rcO2) during SSC can provide staff with real-time reassurance of physiological stability during SSC, even in very premature newborns receiving respiratory support (19, 35, 40). Specialized chairs for SSC, mirrors that allow parents to observe the baby's face, and supportive devices such as slings and restraint systems for tubes and cables may improve perceived safety, ergonomics, and comfort during prolonged SSC sessions. However, evidence remains limited regarding which interventions primarily increase perceived safety vs. actual safety, and which are truly effective in changing clinical practice (17, 33, 41, 50).

Emerging digital tools, including mobile applications and SSC tracking platforms, may support parent education and encourage SSC participation by helping families record and visualize SSC duration over time. Some implementation studies suggest that simply measuring and visualizing their own data can increase SSC frequency and duration. Although evidence remains limited, these tools may represent promising strategies to support SSC implementation and parental engagement (78, 79).

### 4.3 Transformation of professional roles in the implementation of skin-to-skin contact

Given the strong evidence supporting SSC in neonatal care, its implementation should be considered a quality standard rather than an optional practice. Institutional commitment is essential to ensure its sustainable integration into routine care (13, 24, 52, 63). Establishing national or institutional targets compels organizations to prioritize SSC at the same level as other critical medical interventions. Support from hospital administration is critical to providing the necessary resources, including dedicated time and staffing. Although nurse-to-patient ratios of 1:2 are considered standard in many high-income NICUs, more intensive staffing and coordinated teams of at least two trained professionals may be required during SSC transfers of critically ill infants (9, 13, 14, 52, 63, 80).

The designation of “family-centered care and SSC advocates” or “champions” may facilitate cultural change within the team (that is, professionals who actively promote and support SSC implementation within the unit, acting as drivers of change). These leaders can mentor less experienced professionals, normalize SSC in daily practice and foster intrinsic motivation among staff (12, 24, 46, 52). Institutional recognition of neurodevelopmental and family-centered care activities may further reinforce professional engagement (14, 52).

To reduce the anxiety of professionals working under the observation of families, it is necessary to shift the paradigm from “visiting” to “active collaboration” (12, 63). Family collaboration programs such as Family Integrated Care (FICare) or Close Collaboration with Parents, and developmental care approaches such as NIDCAP, may support this transition by encouraging professionals to act as mentors and facilitators rather than sole care providers (12, 77). Although initial implementation of these models requires time and adaptation, evidence suggests that empowering competent parents and progressively increasing their involvement can reduce the demand for care and staff stress in the long term (12, 14, 31, 59, 63, 75, 77). Furthermore, facilitating SSC even in critical or end-of-life situations may increase professional satisfaction and strengthen the therapeutic relationship between staff and families (81, 82).

### 4.4 Empowering families: strategies to overcome sociocultural and personal barriers

To achieve an effective transition to parents becoming active caregivers for their children, neonatal units must adopt a multidimensional approach that addresses psychological, cultural, linguistic, and socioeconomic difficulties. This requires not only facilitating parental presence through some of the measures previously discussed but also providing support to families so they can integrate with confidence, understanding, and emotional security into the NICU environment.

However, the optimal ways to adapt SSC implementation strategies to vulnerable families remain poorly understood.

#### 4.4.1 Personalized communication

Communication should be individualized, culturally sensitive, and focused on emotional connection rather than solely on information delivery (59, 81). Simple strategies such as using the infant's name, maintaining eye-level communication, acknowledging parents as primary caregivers, and providing clear explanations regarding SSC may help reduce parental anxiety and strengthen caregiver confidence (59, 81).

Information regarding SSC should highlight not only the short-term benefits but also the long-term neurodevelopmental benefits, which are of great interest to families (15, 17). Educational videos, visual resources, anonymous question platforms, and family-designed educational materials may further improve parental understanding, communication, and engagement in SSC (41, 83).

Because some families may experience difficulties expressing concerns or asking questions, healthcare professionals should actively promote open and supportive communication environments. Involving experienced or “referential” parents in the development of educational resources may also help improve the relevance, accessibility, and acceptance of family guidance tools (4, 41, 66).

#### 4.4.2 Addressing cultural and linguistic barriers

Communication strategies should also be culturally adapted and accessible to families with limited language proficiency or literacy. Multilingual educational tools, audiovisual resources, interpreters, and cultural mediators may help reduce inequalities in access to SSC-related information and facilitate communication between families and healthcare professionals (57, 59, 83–86).

Healthcare professionals should also remain aware of cultural norms that may influence parental participation, communication styles, and perceptions of authority, in order to avoid misinterpreting silence or reduced questioning as lack of interest (57, 59, 60, 87–89).

#### 4.4.3 Parental care: integrating maternal health and parental well-being into the initiation of skin-to-skin contact

Parental physical and emotional well-being strongly influence SSC initiation and continuity. Postpartum pain, cesarean recovery, maternal illness, psychological distress, and trauma-related responses may reduce parental presence and participation in SSC, particularly in highly medicalized NICU environments that may contribute to feelings of loss of the parental role (1, 18, 24, 53, 54, 90–93).

Given that parental anxiety, depression, and trauma-related responses may directly reduce SSC participation through avoidance behaviors and fear of harming the infant, access to psychological and psychosocial support should be considered an essential component of SSC implementation. Trauma-informed approaches, collaboration with perinatal mental health professionals, and nonjudgmental environments may be particularly important for families experiencing mental health disorders, substance use disorders, previous traumatic experiences, or significant psychosocial vulnerability (4, 17, 61, 62, 94–96).

Peer-support initiatives involving experienced or “referential” parents may complement professional psychological support by normalizing emotional responses, strengthening parental confidence, and facilitating adaptation to the NICU environment (4, 17, 83, 94). Models such as Mother-Newborn Couplet Care may further support SSC implementation by reducing separation, promoting bonding, and reinforcing parents in their role as primary caregivers (91–93).

Fathers and non-birthing caregivers should also be actively incorporated into SSC implementation strategies. Supporting their participation may help maintain SSC continuity, strengthen bonding, and reduce the disruption of the parental role during NICU admission. Their role will be particularly important during periods in which mothers are temporarily unavailable due to postoperative recovery, medical complications, or psychological distress (24, 41, 70, 71).

#### 4.4.4 Financial and logistical support for families

Neonatal units should implement specific strategies in collaboration with social services and institutional support systems. Practical support measures such as transportation assistance, free or subsidized parking, accommodation near the hospital, meal support, and on-site childcare for siblings may increase parental presence and, therefore, opportunities for more parental involvement (18, 68, 70, 89).

Temporary financial assistance to low-income families may represent a promising strategy, although evidence regarding cost-effectiveness remains limited. Providing financial support could increase parental presence and SSC, thus facilitating faster weight gain and greater physiological stability—key criteria for discharge. These interventions could also generate savings for the healthcare system by optimizing the costs of prolonged intensive care (13, 31, 53, 68, 69, 89–91).

## 5 Discussion

### 5.1 Interpretation and implications for practice

Despite the strong evidence supporting SSC, its implementation remains inconsistent across NICUs worldwide. This review suggests that the gap between evidence and practice is not primarily explained by lack of evidence regarding clinical benefit, but rather by the interaction between organizational culture, professional perceptions of safety, parental presence, and institutional resources.

One of the main findings emerging from this review is that SSC implementation depends less on isolated interventions than on the ability of neonatal units to integrate SSC into the routine culture of care. In this context, professional awareness and training, and parental presence appear to be two central determinants of successful implementation.

The review also highlights several unresolved challenges in SSC implementation across different NICU models. While SFRs may facilitate parental presence and SSC in some settings, current evidence regarding their long-term neurodevelopmental impact remains mixed, particularly in infants with limited parental presence. Importantly, SSC implementation should not depend exclusively on high-resource architectural redesign or increased staffing, as this may further increase inequities in access to SSC across healthcare settings. Several low-cost and adaptable interventions —including standardized protocols, staff training, workflow optimization, family guidance tools, ergonomic furniture, and privacy adaptations— may facilitate SSC across open-bay, hybrid, and SFR NICU models. Similarly, although increasing evidence supports SSC even in highly fragile infants, professional concerns regarding safety continue to limit implementation in many settings, highlighting the need for standardized protocols, training, and organizational support.

This review has several limitations. As a narrative review, it was not designed to systematically evaluate the quality of evidence or quantify the effect of specific interventions. In addition, many implementation studies are heterogeneous and context-dependent, limiting direct comparisons across settings. However, this approach allowed integration of clinical, organizational, psychosocial, and equity-related dimensions that are often analyzed separately in the literature.

Importantly, not all proposed implementation strategies are currently supported by the same level of evidence, and several approaches discussed in this review remain exploratory or context dependent.

### 5.2 What can NICUs do now?

While some implementation strategies require significant structural investment, many practical actions can be adopted immediately in different NICU settings.

Standardized SSC eligibility and transfer protocols, simulation-based staff training, designation of SSC champions, and structured family guidance programs may help reduce variability in practice without requiring extensive financial resources. Similarly, low-cost environmental adaptations, such as ergonomic chairs, privacy screens, and family-friendly spaces, can facilitate parental presence and SSC in both high- and low-resource settings.

Promoting consistent communication among healthcare professionals and families, and actively engaging parents as primary caregivers should also be considered core implementation priorities. In this regard, trauma-based approaches, psychosocial support and peer-support programs can help families, particularly those experiencing anxiety, depression, or social vulnerability.

### 5.3 Knowledge gaps and future research priorities

Several important knowledge gaps remain regarding SSC implementation in highly fragile neonatal populations, including infants at the limit of viability, those receiving HFOV, and infants with complex devices such as pleural drains or umbilical arterial catheters.

Further research is also needed to better understand the long-term impact of different organizational and architectural models, particularly regarding equity, sensory stimulation, and parental presence.

In addition, evidence remains limited regarding the sustainability, scalability, and cost-effectiveness of many implementation strategies, especially in low-resource settings. Future implementation-focused research should prioritize pragmatic and multicenter approaches capable of evaluating interventions under real-world clinical conditions.

## 6 Conclusion

SSC should be considered a fundamental component of neuroprotective and infant- and family-centered developmental care. Although important barriers to its implementation persist, promoting continuous parental presence and fostering a shared understanding among healthcare professionals that SSC is a safe and stabilizing intervention are essential for its successful integration into routine care.

Despite major differences in resources across healthcare settings, many low-cost strategies may help reduce inequities and support SSC implementation across diverse NICU contexts. Future research should focus on sustainable and scalable implementation strategies, particularly for the most fragile neonatal populations.
